# Draft Genome Sequences of *Microbacterium hominis* LCDC-84-0209^T^ Isolated from a Human Lung Aspirate and *Microbacterium laevaniformans* LCDC 91-0039 Isolated from a Human Blood Culture

**DOI:** 10.1128/genomeA.00989-16

**Published:** 2016-09-15

**Authors:** Anne-Marie Bernier, Kathryn Bernard

**Affiliations:** a Department of Biology, Université de Saint-Boniface, Winnipeg, Canada; b National Microbiology Laboratory-CSCHAH Site, Public Health Agency of Canada, Winnipeg, Canada; c Department of Medical Microbiology, University of Manitoba, Winnipeg, Canada

## Abstract

Draft genomes for *Microbacterium hominis* 84-0209^T^ and *M. laevaniformans* 91-0039 were studied. Genome sizes (bps, [G+C contents]) were 3,506,522 (70.96%) and 2,999,965 (69.51%), respectively. Annotation revealed: (*M. hominis*) three rRNA sequences, 45 tRNA genes, and 3,218 coding sequences; (*M. laevaniformans*) three rRNA sequences, 49 tRNA genes, and 2,874 coding sequences.

## GENOME ANNOUNCEMENT

Members of the genus *Microbacterium* are environmentally derived, Gram stain-positive bacilli which infrequently cause opportunistic infections in humans (1). Some of these coryneforms were originally designated under provisional names CDC Coryneform Group A-4 and A-5 in the 1980s (2). LCDC 84-0209^T^ (CDC group A-4) and LCDC 91-0039 (CDC group A-5) were shared by Health Canada’s Laboratory Centre for Disease Control (LCDC, Ottawa, Ontario, now the National Microbiology Laboratory [NML] in Winnipeg, Manitoba) with European and Japanese colleagues as part of an effort to assign provisionally named bacteria to validly published genera. Many CDC group A-4 and A-5 isolates were assigned to the genus *Microbacterium* in 1995 after 16S rRNA gene sequencing analysis (3). In 1998, Takeuchi and Hatano added six new species to this genus, including *M. hominis* (LCDC 84-0209^T^ = IFO [NBRC] 15708^T^ = CIP 105731^T^ = DSM 12509^T^ = JCM 12413^T^ = VKM Ac-2081^T^, a human lung aspirate isolate) as well as *M. laevaniformans* LCDC 91-0039 (=IFO [NBRC] 15709, a blood culture isolate) (4). In this study, we describe features derived from the whole-genome sequencing of LCDC 84-0209^T^ and LCDC 91-0039.

DNA was extracted using the TruseqR DNA HT sample preparation kit. Paired-end whole-genome shotgun libraries were constructed using TruseqR Nano DNA HT library preparation kit and mate-pair libraries for each strain were prepared using the Nextera mate-pair sample preparation kit (Illumina). Sequencing was performed using a MiSeq sequencer (Illumina).

Sequencing of paired-end libraries for *M. hominis* and *M. laevaniformans* generated 2,296,026 and 1,583,940 reads; mate-pair libraries gave rise to 2,272,076 reads and 2,768,080 reads, respectively. Overlapping paired-end reads were merged using FLASH (fast length adjustment of short reads) (5) to produce longer single reads that were subsequently assembled with paired-end reads, using SPAdes genome assembler (St. Petersburg genome assembler, version 3.5.0) using k-mer values of 21 to 127 (6).

Assembled contigs were filtered to remove short (≤500 pb) and repeat sequences; mate-pair reads were added to contigs using Sspace (v2.0.0) to produce scaffolds (7). This resulted in draft genomes of 3,506,522 bp (73 contigs in 12 scaffolds) and 2,999,965 bp (59 contigs in 10 scaffolds), with 70.96% and 69.51% G+C content for *M. hominis* and *M. laevaniformans*, respectively. Coverage was 115× and 148×, respectively. Annotation of *M. hominis* using the Prokaryotic Genome Automatic Annotation Pipeline (PGAAP, NCBI) predicted three rRNA sequences, 45 tRNA genes, and 3,218 coding sequences. Data was highly consistent (>99% symmetric identity [SI]) with an unpublished genome of *M. hominis* NBRC 15708^T^ (Genbank [GB] accession no. NZ_BCW100000000.1); both genomes were not closely related (63% SI) to GB NZ_JWSZ00000000.1. Annotation of *M. laevaniformans* using Prokka (8) revealed three rRNA sequences, 49 tRNA genes, and 2,874 coding sequences. Genome analysis using PHAST (PHAge Search Tool [9]) revealed that *M. hominis* harbors one intact phage of 37.3 Kb with homology to *Mycobacterium* phage NiaZeal.

### Accession number(s).

Draft whole-genome projects for *M. hominis* LCDC 84-0209^T^ and *M. laevaniformis* LCDC 91-0039 have been deposited at DDBJ/EMBL/GenBank under accession no. LRYC00000000 and LRAD00000000.
